# PediLoc^®^ blade plate yields faster consolidation rates than LCP pediatric condylar plate in distal femoral osteotomies: a retrospective comparison and description of surgical technique

**DOI:** 10.1186/s10195-026-00946-2

**Published:** 2026-06-24

**Authors:** Samuel Tschudi, Cyrill Delay, Britta Krautwurst, Domenic Grisch, Thomas Dreher

**Affiliations:** 1 Department of Pediatric Orthopaedics and Traumatology, Children’s University Hospital Zurich, Lenggstrasse 30, 8008 Zurich, Switzerland; 2 Department of Orthopaedics and Traumatology, Hospital of Buelach, Buelach, Switzerland; 3 Department of Children’s Orthopaedics and Traumatology, Balgrist University Hospital Zurich, Zurich, Switzerland

**Keywords:** Locking plate system, Blade plate, Distal femoral osteotomy, Deformities, Multidimensional osteotomies, Paediatric orthopaedics, Surgical technique

## Abstract

**Background:**

The PediLoc^®^ Locking Cannulated Blade Plate System was originally designed for proximal femoral osteotomies but has also been used for multidimensional correction of distal femoral deformities. The present study delineates the surgical technique and undertakes a retrospective analysis of the consolidation rates of distal femoral osteotomies performed using the aforementioned system, comparing them with those performed using the DePuy Synthes^®^ 90° LCP Pediatric Condylar Plate. We hypothesized that the semi-rigid design of the blade plate results in faster osteotomy healing rates compared with other locking screw plate systems.

**Materials and methods:**

This retrospective single-centre study included patients who underwent distal femoral osteotomy with the PediLoc^®^ Blade Plate (Blade Plate) and the DePuy Synthes^®^ 90° LCP Pediatric Condylar Plate (90° LCP-PCP) at the University Children’s Hospital Zurich (2020–2024). Consolidation rates were assessed using "Regenerating Bone Defects Using New Biomedical Engineering" (REBORNE), "Radiographic Union Score for Tibial Fractures" (RUST) and modified RUST (mRUST) scores at 6 weeks and 3 months postoperatively and compared using using Wilcoxon signed-rank test. For the comparison between both intervention groups, the unpaired *t*-test was used for normally distributed data and the Mann–Whitney *U* test for non-normally distributed and ordinal data. For all tests, values of *p* < 0.05 were considered statistically significant.

Geometric planes of osteotomy were analysed, and a stepwise correction technique was described.

**Results:**

A total of 111 osteotomies with complete radiographic follow-up were analysed (77 Blade Plate [69.4%], 34 90° LCP-PCP [30.6%]). At 6 weeks, early consolidation (RUST score ≥ 10) was observed in 39% of Blade Plate cases versus 6% in the 90° LCP group. By 3 months, consolidation reached 91% in the Blade Plate group and 70% in the 90° LCP-PCP group. One Blade Plate case was excluded due to an implant-associated intraoperative fracture.

**Conclusions:**

The PediLoc^®^ Blade Plate facilitates stable, multidirectional correction of distal femoral deformities and achieves earlier radiographic consolidation of osteotomies compared with the 90° LCP-PCP without increased complication rates. Since faster consolidation was observed only in the RUST scores, the clinical relevance of this finding remains to be determined through prospective randomized controlled trials.

**Level of evidence:**

Level III, retrospective comparative study.

**Trial registration:**

Clinicaltrials.gov, 2025-0000, registration date 14 April 2025, retrospectively registered, https://register.clinicaltrials.gov/prs/beta/records

## Introduction

The PediLoc^®^ Locking Cannulated Blade Plate System (Blade Plate) is primarily intended for proximal femoral osteotomies. However, due to its design, it is a tool of great practical value for one-, two- or three-dimensional deformity correction at the distal femur [1, 2]. Common indications are knee flexion contracture which endures despite rigorous conservative treatment, rotational deformities and varus or valgus deformities of the distal femur that cause pain, discomfort or gait disturbances and are not amenable for conservative treatment. Especially in multidimensional deformities, its design offers easier control over the osteotomy’s planes [2].

Due to its semi-rigid design, the Blade Plate provides high stability while permitting a certain degree of micromovement at the osteotomy site. In biomechanical models, this results in accelerated callus formation and osteotomy healing [3–6]. Recent case series have confirmed the clinical application of the Blade Plate for distal femoral osteotomies, showing safe and reliable correction with low complication rates in paediatric cohorts up to skeletal maturity [7–10]. Moreover, the use of cannulated blade-plate systems has been associated with facilitated surgical technique and enhanced fragment control [11] during multidimensional corrections, as reported in a 2020 biomechanical evaluation [12].

Based on these biomechanical properties, it was hypothesized that the semi-rigid blade plate design would result in faster osteotomy healing rates compared with conventional locking screw plate systems.

The objectives of this study were, firstly, to provide a detailed description of the surgical technique employed; secondly, to demonstrate the efficacy of the Blade Plate in enabling a stepwise correction of individual deformity planes; and thirdly, to perform a retrospective comparison of consolidation rates at defined time points with those of the Synthes^®^ 90° LCP Pediatric Condylar Plate (90° LCP-PCP).

## Materials and methods

### Methodology

The present retrospective single-centre study investigated the electronic medical records of patients who underwent distal femoral osteotomy with the Blade Plate system and the 90° LCP-PCP at the University Children’s Hospital Zurich between 2020 and 2024. The data used in this study were collected during routine clinical practice between 2020 and 2024.

The objective of this study was to analyse various parameters to evaluate the surgical procedure and healing processes. The consolidation rates of the osteotomies were using the "Regenerating Bone Defects Using New Biomedical Engineering" (REBORNE) [13, 14], "Radiographic Union Score for Tibial Fractures (RUST) [15–18] and modified RUST (mRUST) [16, 17] scores on serial X-ray images, including intraoperative imaging as well as postoperative imaging at 6 weeks and 3 months. Furthermore, a comprehensive analysis of surgical reports was conducted to ascertain the geometric planes of the osteotomy, incorporating rotational corrections, varus/valgus corrections, flexion or extension adjustments and multidimensional corrections.

The study encompassed all patients who underwent distal femoral osteotomy with the Blade Plate or the 90° LCP-PCP during the specified period. Patients were excluded if they received alternative fixation systems, underwent osteotomies at different anatomical sites, experienced intraoperative complications resulting in implant failure, lacked radiographic follow-up at 6 weeks or 3 months postoperatively, or had previously undergone surgery at the same anatomical site.

The deformity assessment comprised the following elements:

- Measurement of mechanical lateral distal femur angle (mLDFA) and mechanical medial proximal tibial angle (mMPTA) in a preoperative long-leg standing radiograph.
- Rotational assessment of femoral antetorsion and tibial external rotation in a rotational magnetic resonance imaging (MRI).
- Pre- and intraoperative clinical assessment of passive extension deficit.

The postoperative management protocol was identical for both groups. Partial weight-bearing of 10–15 kg was permitted for 6 weeks, provided that the patient was ambulatory and cognitively able to comply with weight-bearing restrictions. Thereafter, full weight-bearing was allowed as tolerated if bone consolidation was sufficient. Supervised physical therapy was prescribed for a total duration of 3 months postoperatively. The initial phase focused on restoring range of motion and mitigating muscle atrophy associated with the 6-week period of partial weight-bearing. This was followed by a progressive rehabilitation phase lasting from 6 weeks to 3 months postoperatively, emphasizing strength recovery and gait normalization.

### Statistical analysis

The data were tested on normal distribution. Normally distributed data were presented as mean (standard deviation [SD]) and non-normally distributed data as medians and interquartile range (IQR). For the comparison between both intervention groups, the unpaired *t*-test was used for normally distributed data and the Mann–Whitney *U* test for non-normally distributed and ordinal data. The pre–post comparison of non-normally distributed data was performed using the Wilcoxon signed-rank test. For all tests, values of *p* < 0.05 were considered statistically significant. Statistical analyses were performed with SPSS Statistics V.30 (SPSS Inc., IBM Company, Chicago, IL, USA).

### Ethical approval and consent to participate

The present study was conducted in accordance with rigorous ethical guidelines. The study was approved by the local ethics committees (Cantonal Ethics Committee of Zurich project number 2025-00007) prior to the commencement of the analysis. The analysis was conducted anonymously, in strict accordance with the prevailing data protection regulations. The inclusion of patients in this study was contingent upon the prior completion and signature of a general consent form prior to undergoing surgical intervention.

### Description of surgical technique

#### Indications

**Fig. 1:**
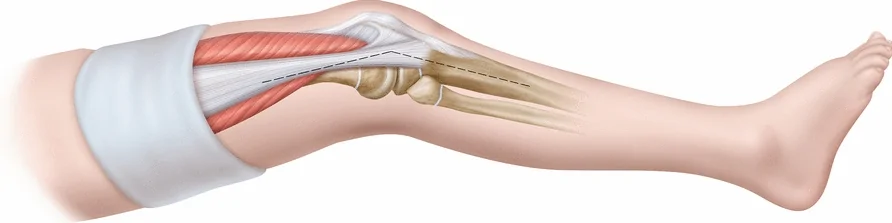
Evaluation of preoperative flexion contracture

- Multidimensional deformity correction in children and adolescents with or without neurogenic disorders (e.g. cerebral palsy).
- Fixed knee flexion contracture (Fig. 1), with or without additional rotational and/or varus/valgus deformity of the distal femur, in children and adolescents with or without neurogenic disorders (e.g. cerebral palsy).

#### Surgical approach

##### Preparations

A thorough three-dimensional deformity analysis is imperative for the successful correction of the deformity. This encompasses conventional radiographs in two planes, rotational computed tomography (CT) or magnetic resonance imaging (MRI) scans of both legs, as well as CT or MRI of the affected area. In the event of its presence, the degree of knee flexion contracture is measured and documented prior to surgery (Fig. 1).

Key point

- Measure flexion contracture prior to inflating the tourniquet, as this may result in tightening of the hamstring muscles and subsequently a mimicry of a greater amount of flexion contracture.

##### Approach

**Fig. 2:**
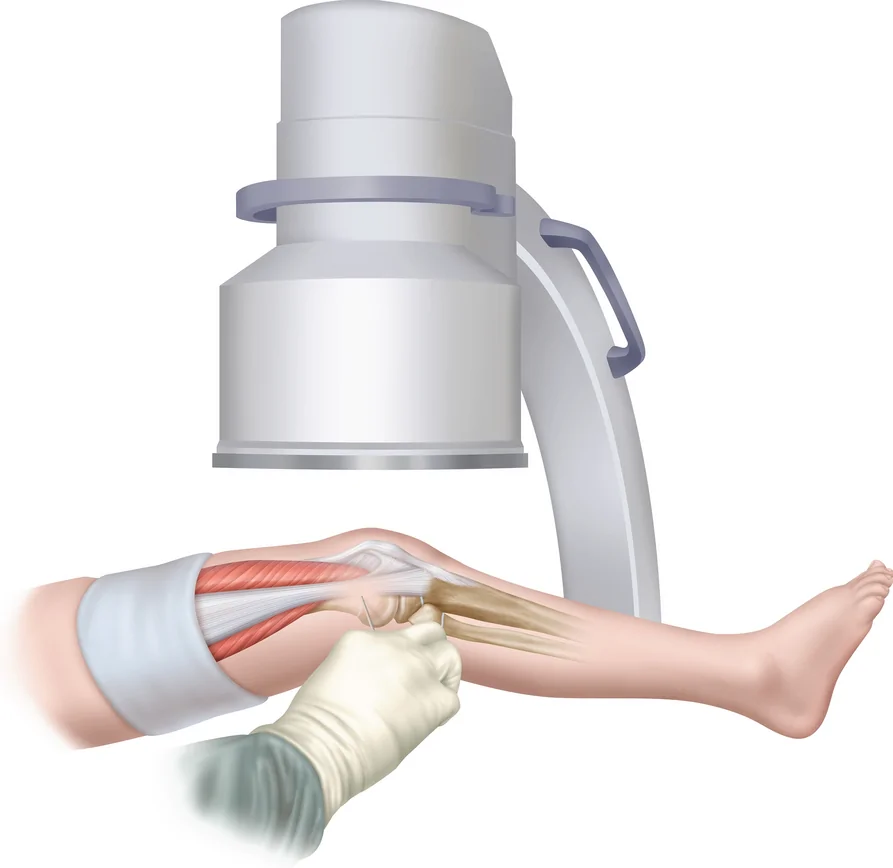
Radiological determination of osteotomy site

The anatomical landmarks should be palpated and marked, the level of the distal femoral physis identified and the desired height of the osteotomy defined using fluoroscopy (Fig. 2).

**Fig. 3:**
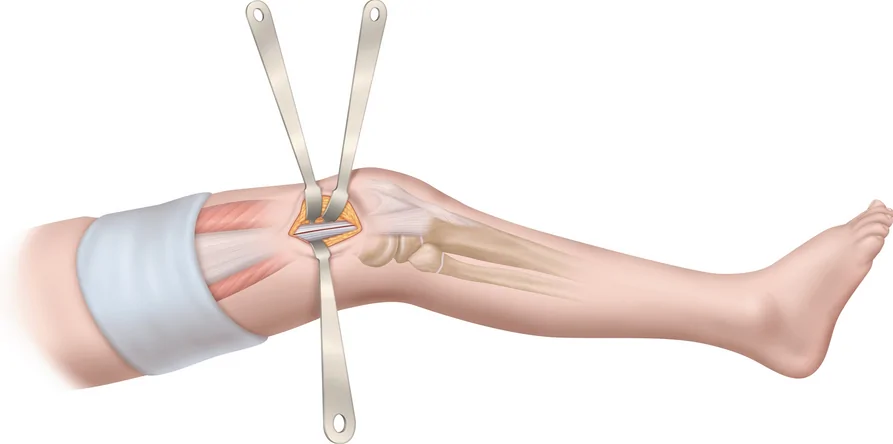
Approach: iliotibial and split

Make a longitudinal incision parallel to the distal femur, starting 0.5–1 cm above the marked physis and extending approximately 8 cm proximally (Fig. 3).

**Fig. 4:**
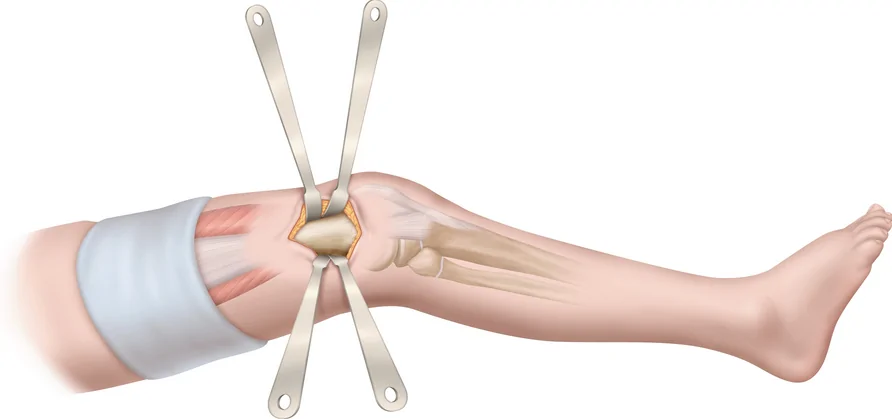
Approach: placement of Hohmann levers

Continue the sub-vastus approach to expose the distal femur. Insert four Hohmann retractors – two anterior and two posterior (Fig. 4).

Key point

- A minimum distance of 10 mm between the distal end of the plate and the physis is recommended. Any damage to the physis must be avoided.

##### Prepare correction (coronal and sagittal plane)

**Fig. 5:**
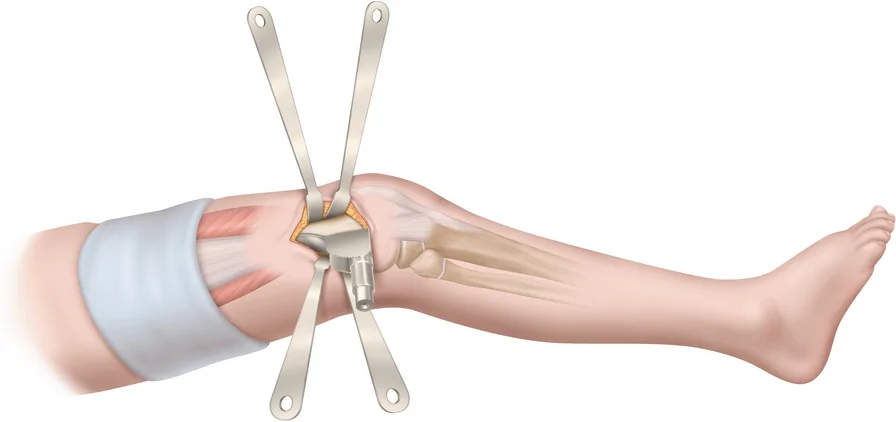
Placement of adjustable angled wire guide determine varus or valgus correction

Position the guide wire at the desired angle to achieve varus or valgus correction. This step may be supported using the corresponding guide. In the absence of concomitant frontal plane corrections (varus/valgus), the adjustable angled wire guide is set to 90° and positioned onto the distal femoral bone (Fig. 5).

**Fig. 6:**
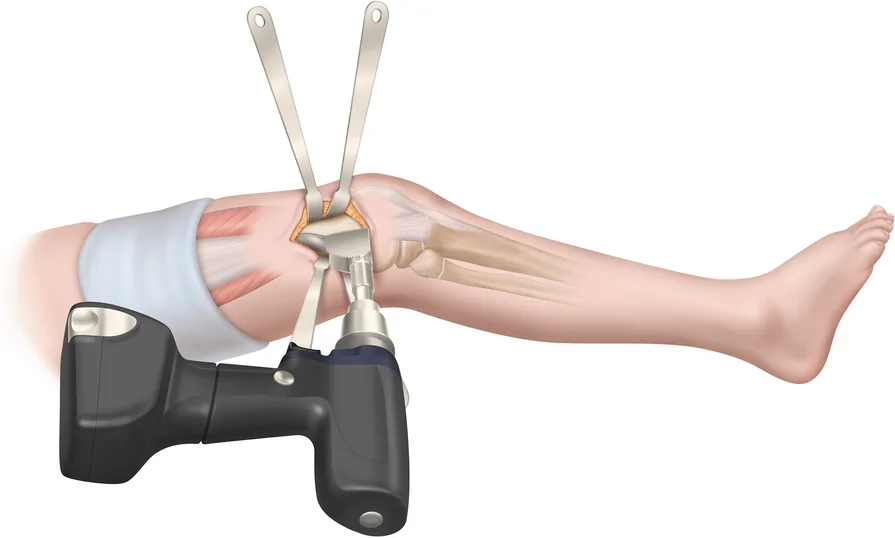
Insert guide wire through the wire guide in determined coronal angle

**Fig. 7:**
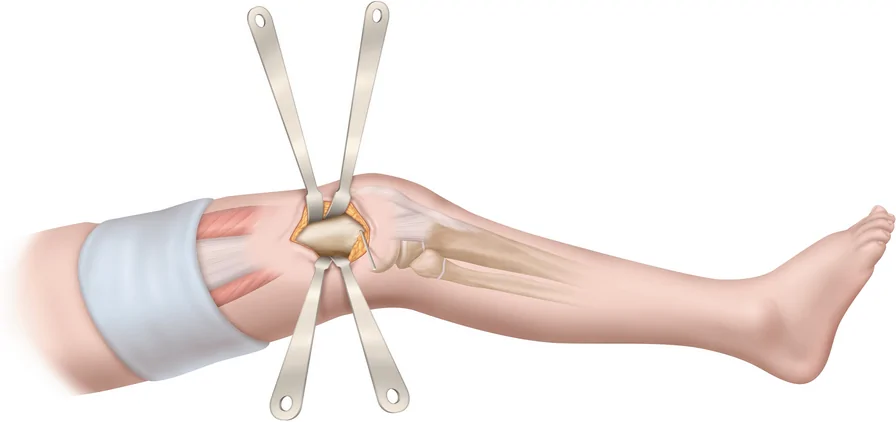
Placed guidewire

Insert the 2.0 mm guide wire under fluoroscopic control until it just perforates the medial cortex (Figs. 6, 7).

**Fig. 8:**
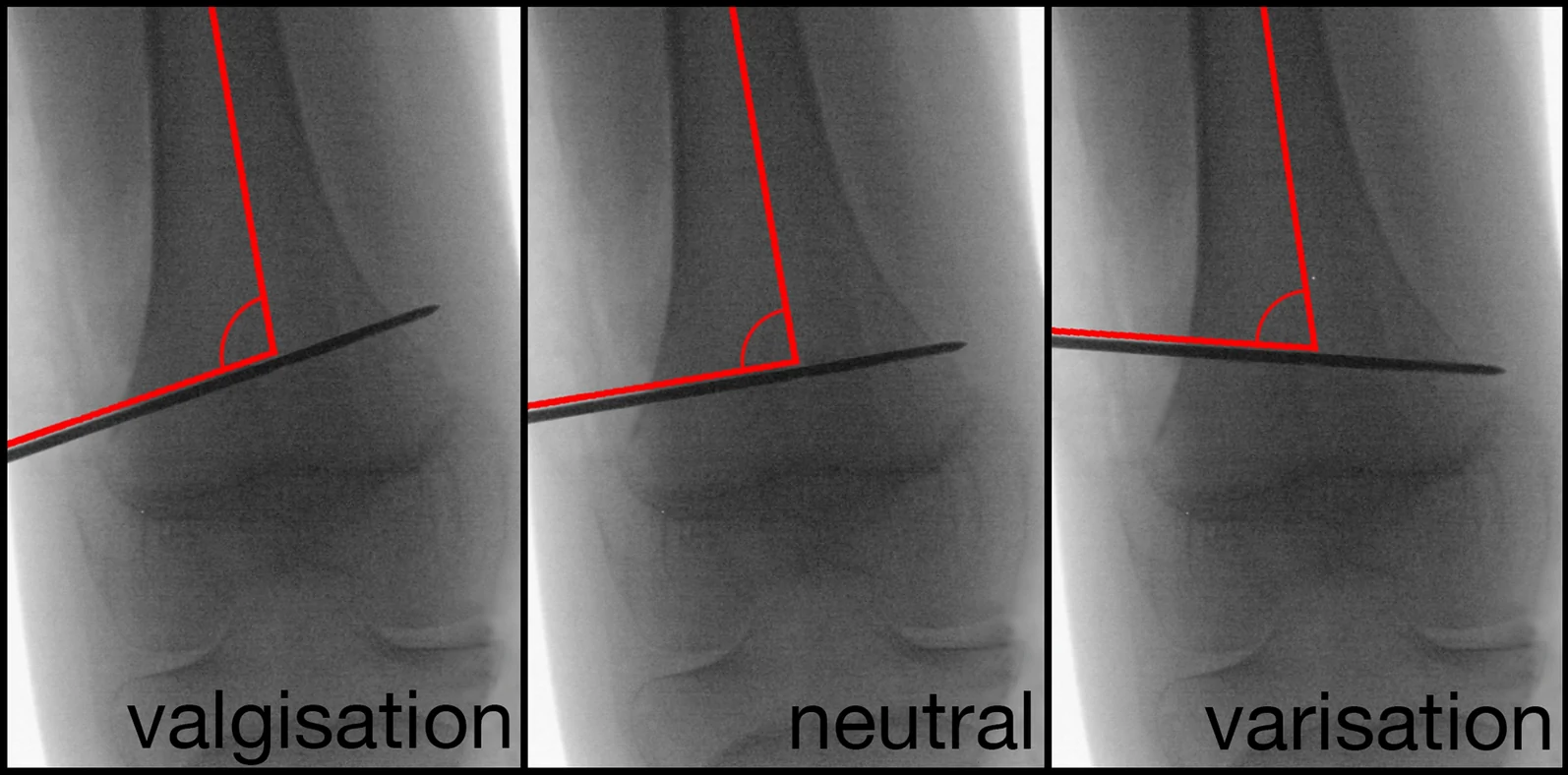
Illustration of coronal plane deformity correction with 2.0 guide wire placed just perforating the medial cortex

If a varus or valgus deformity requires correction, adjust the drill guide to the desired angle using the following rules:

- Varus deformity correction: drill guide angle = plate angle – varus deformity
- Valgus deformity correction: drill guide angle = plate angle + valgus deformity (Fig. 8).

Key point

- In the context of an extension osteotomy, the placement of the wire in an anterior position is crucial to prevent any alteration to the sagittal mechanical axis. In the absence of a sagittal plane correction, the guide wire may be placed centrally in the sagittal plane.

**Fig. 9:**
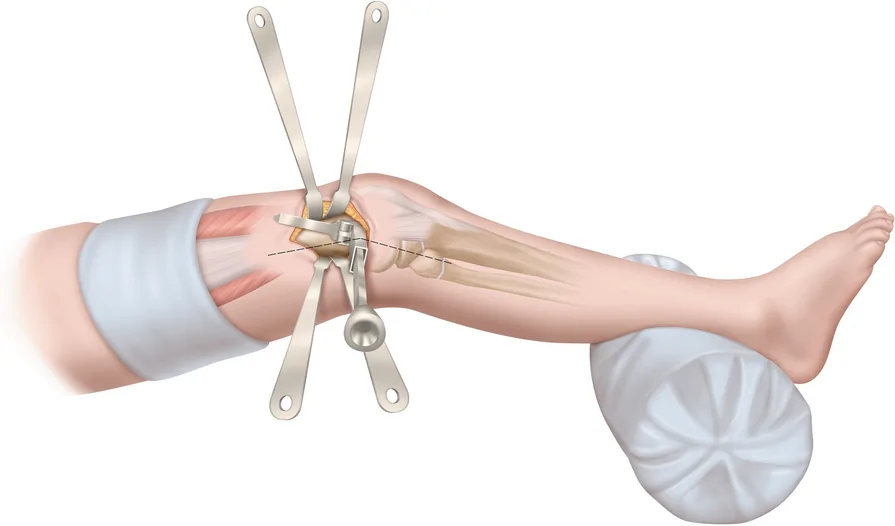
Chisel placement over guide determining sagittal plane correction according to tibial axis in full knee extension

To correct sagittal plane deformities, it is necessary to position the chisel perpendicular to the tibial axis with the knee in full extension prior to its insertion into the distal femur (Fig. 9).

**Fig. 10:**
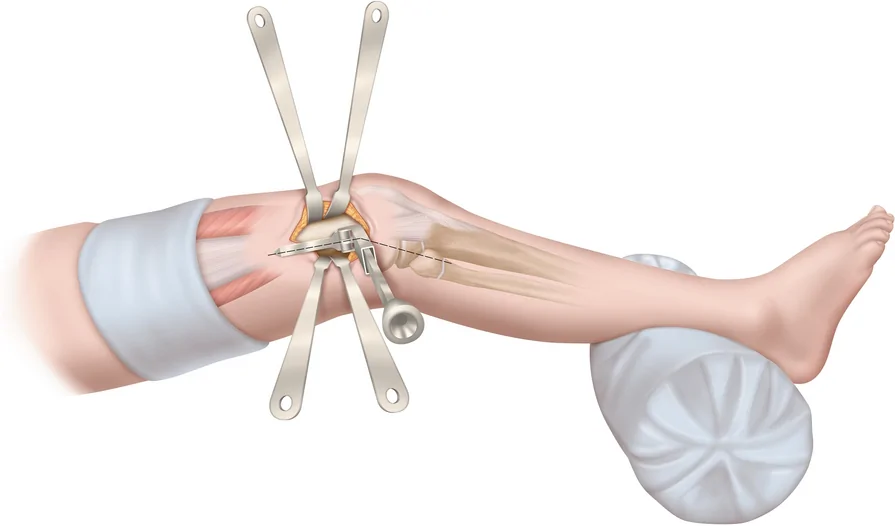
Chisel placement over guide without sagittal plane correction

In cases involving solely rotational or frontal plane correction, with no requirement for sagittal plane correction, the chisel guide is aligned with the femoral axis (Fig. 10).

Before advancing the chisel, drill two to four holes using a 2 mm drill, located just anterior and posterior to the guide wire to prevent fracturing of the femur. Advance the chisel until the medial cortex is reached (Fig. 12).

**Fig. 11:**
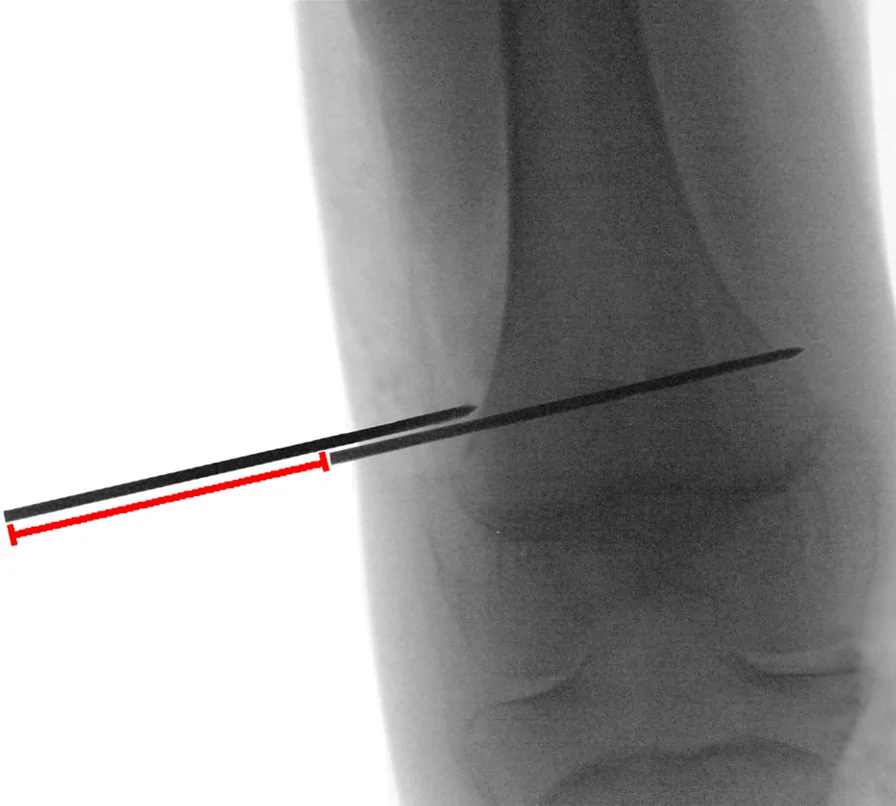
Predetermination of blade length measuring the length of inserted Kirschner wire (k-wire)

Key points

**Fig. 12:**
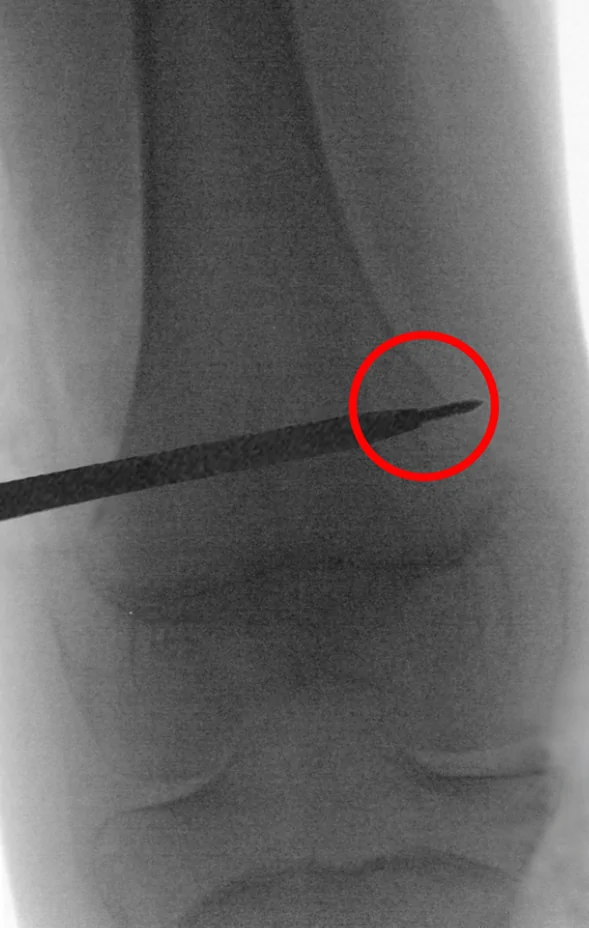
Insertion of the chisel without medial cortex penetration

- The degree of extension correction is determined by the angle of the chisel, which must be perpendicular to the fully extended tibia.
- Avoid penetrating the medial cortex, as this increases the risk of fracture (Fig. 12).

**Fig. 13:**
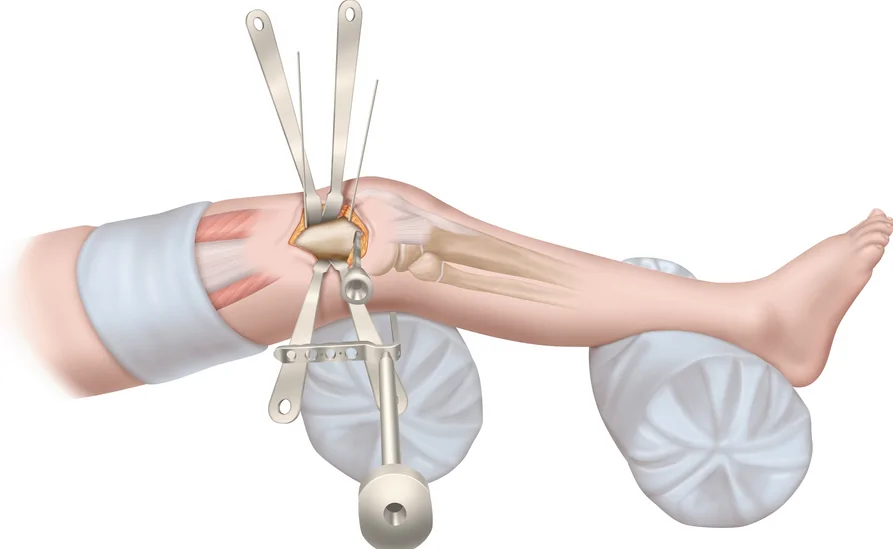
Selected plate attached to inserter

The required blade length should be determined according to the insertion depth of the chisel, which can be read from the sides of the chisel (Fig. 13).

##### Osteotomies

**Fig. 14:**
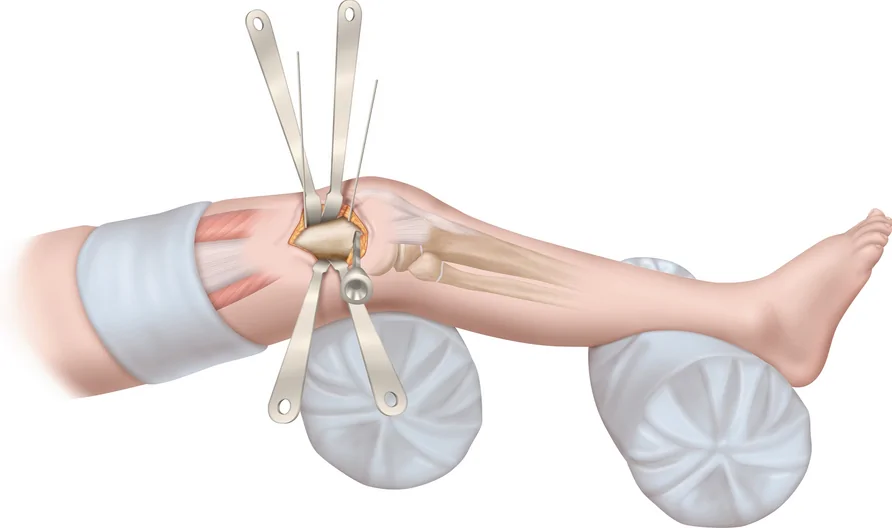
Kirschner wires (k-wires) placed proximal and distal to planned osteotomy to control rotation

Prior to performing the osteotomy (or osteotomies), insert a Kirschner wire on each side of the designated osteotomy to ensure rotational control (Fig. 14).

**Fig. 15:**
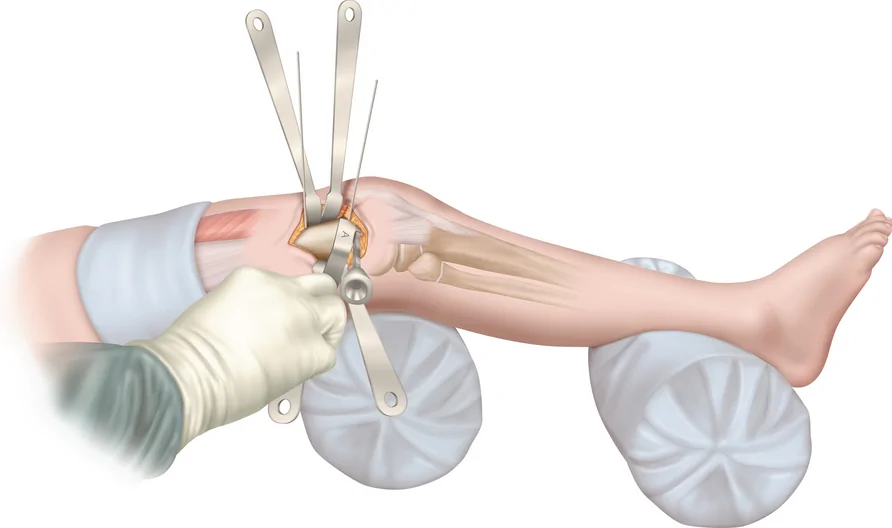
Measuring distance from distal osteotomy to chisel with a gauge instrument

Use the osteotomy gauge instrument to determine the distance from the distal osteotomy to the guide chisel (Fig. 15).

Key points

**Fig. 16:**
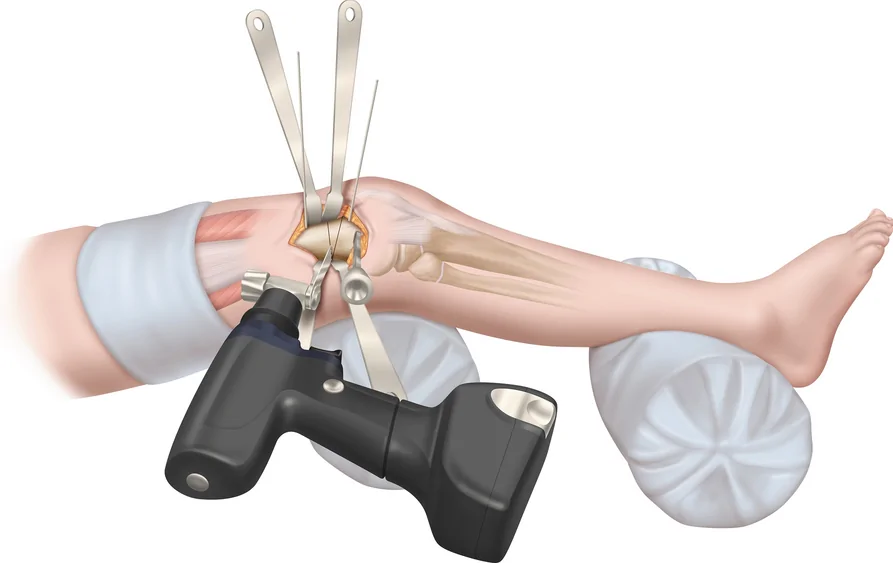
Distal osteotomy perpendicular to the chisel and proximal osteotomy perpendicular to the femoral longitudinal axis

**Fig. 17:**
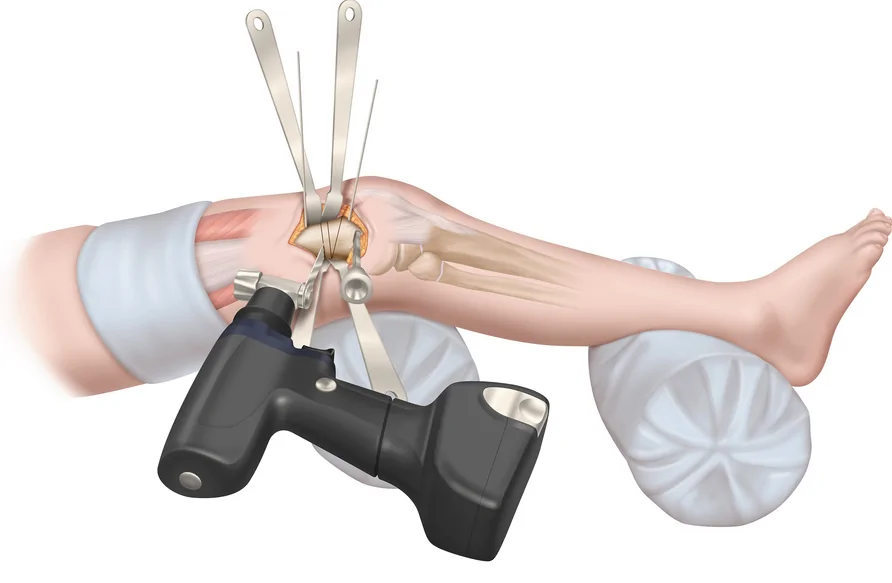
Additional femoral shortening in case of an extension osteotomy

- For sagittal or coronal plane correction, two osteotomies are required.
- The distal osteotomy is performed parallel to the guide chisel in both the coronal and sagittal planes.
- The proximal osteotomy is performed perpendicular to the longitudinal axis of the femur, proximally to the distal cut.
- It is important to recognize that extension osteotomies necessitate a concomitant shortening of approximately 0.5 cm per 10° of extension, a procedure that is requisite to prevent overstretching of neurovascular structures (Figs. 16, 17).

**Fig. 18:**
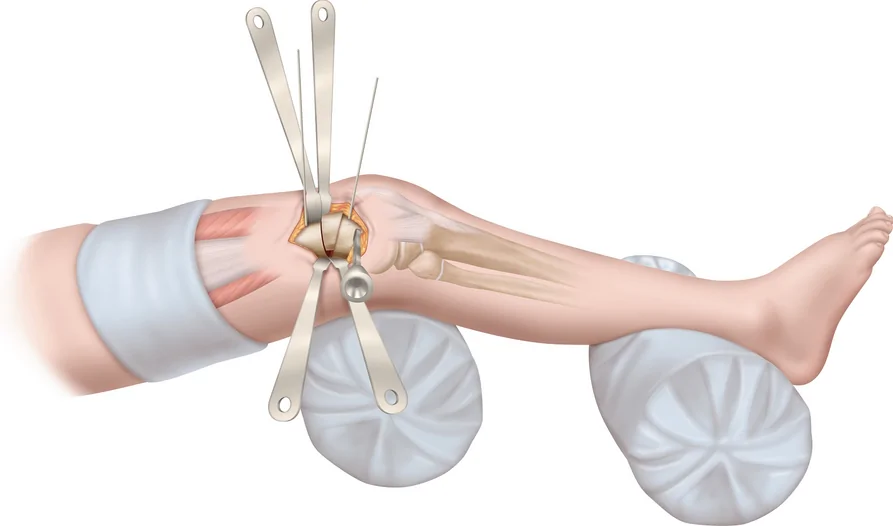
Removal of trapezoid bony wedge

**Fig. 19:**
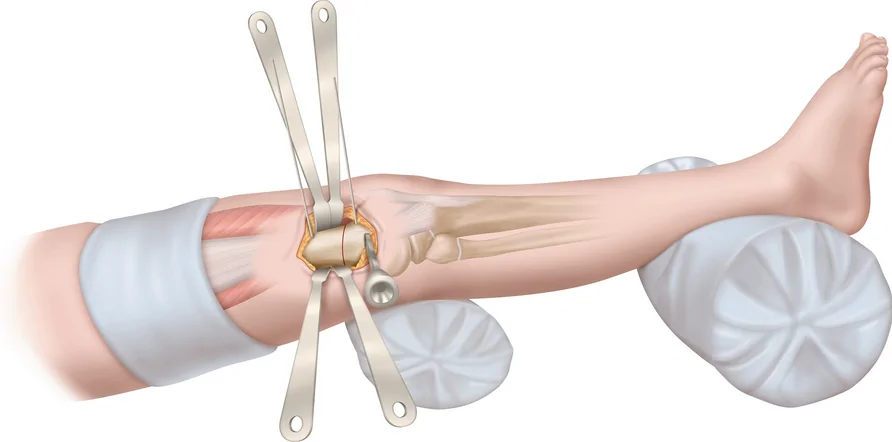
Testing of correction

Once the osteotomies are complete and, if present, the trapezoidal bone wedge has been removed, evaluate correction in all planes (Figs. 18, 19).

Key points

If the deformity is not fully corrected, several factors must be considered:

- Tight soft tissue and neurovascular structures – further femoral shortening or hamstrings lengthening may be necessary.
- Inadequate correction during chisel guide placement – if the residual correction is 10° or less, it may be compensated by placing the plate obliquely on the proximal femoral fragment. If the deficit is greater, re-positioning of the chisel guide is recommended.

##### Plate fixation

The distal fragment is stabilized using reposition forceps, and the plate’s offset is determined depending on the difference in circumference between the two fragments. In cases involving a larger correction, medialization of the proximal fragment relative to the distal fragment may be necessary to maintain the mechanical axis. In such cases, the larger offset variant may be used.

**Fig. 20:**
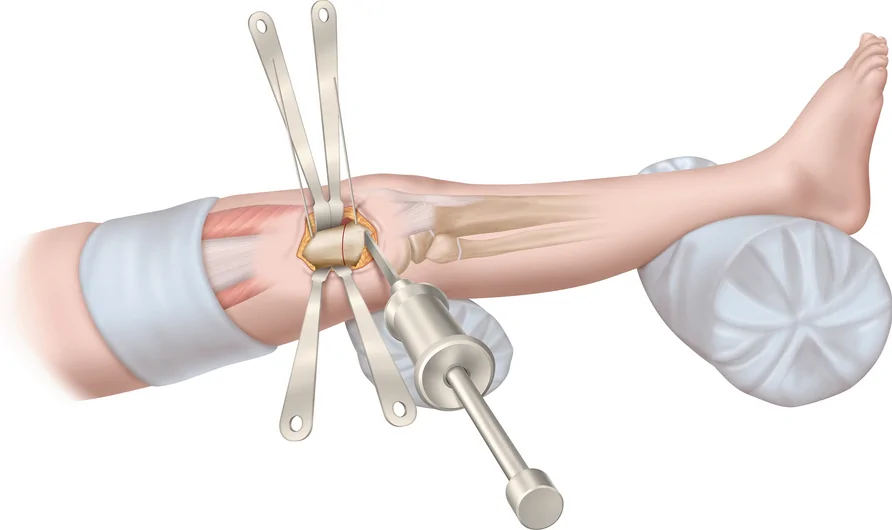
Removing the chisel using the explant device and the sliding hammer

**Fig. 21:**
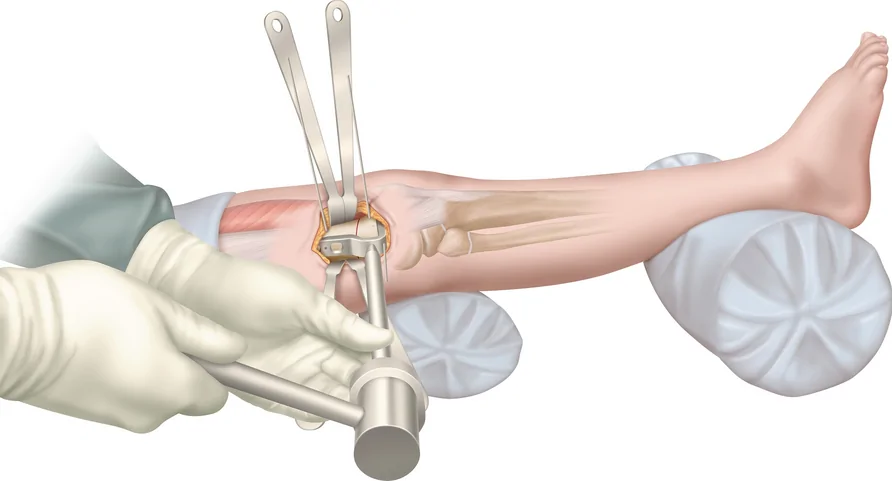
Plate insertion over k-wire in the exact same position as the previously removed chisel

The chisel is removed using the explant device and sliding hammer, and the plate is then inserted over the Kirschner wire (k-wire). It is crucial to insert the blade the exact same angle as the chisel was in before to achieve the planned correction (Figs. 20, 21).

Prior to proximal fixation, adjustment for the desired rotational alignment is required.

**Fig. 22:**
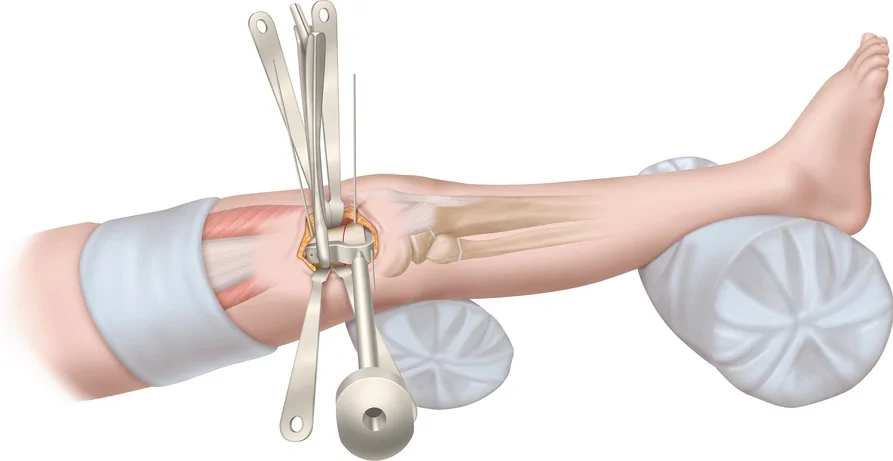
Provisional fixation of the closed osteotomy using the provided bone clamp

**Fig. 23:**
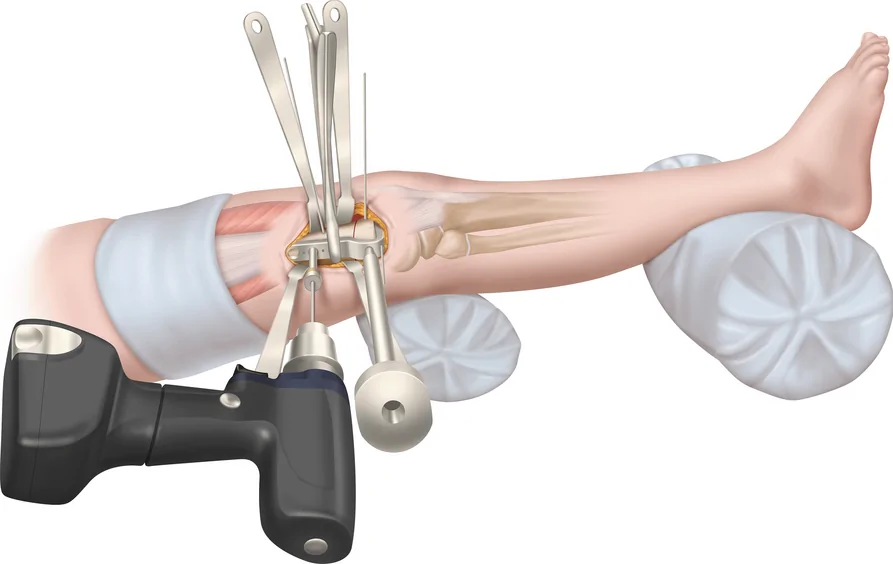
Plate fixed proximally with k-wire(s), testing deformity correction

As indicated by the previously inserted rotational k-wires, the proximal fragment should be preliminary secured to the plate with the clamp provided. The k-wires should then be inserted into angular-stable locking holes through the angular-stable drill guides and reduction cannulas (Figs. 22, 23).

**Fig. 24:**
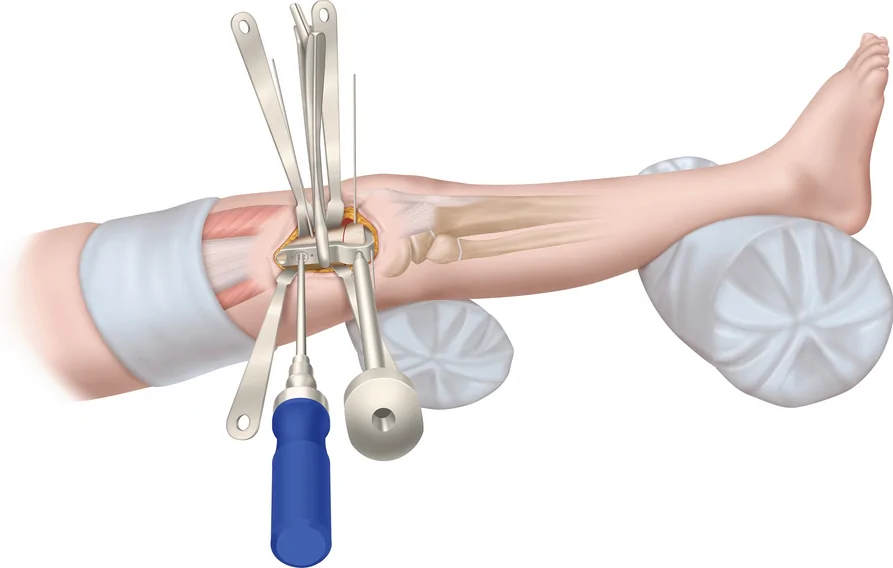
Definitive fixation of the plate proximally

At this stage, the amount of correction can be evaluated. If the desired rotational and extension correction has been achieved, proceed with definitive proximal screw fixation (Fig. 24).

Key points

**Fig. 25:**
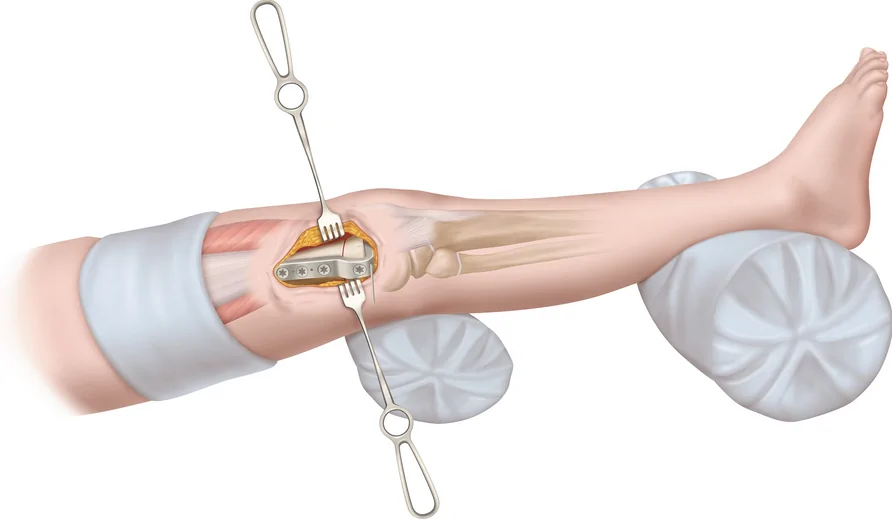
Final clinical evaluation

- If no dynamic compression is intended, begin by inserting locking screws into the locking holes (Fig. 25).
- If dynamic compression is intended, insert cortical screws (Fig. 24) into the dynamic compression slot first. It is imperative to remove the k-wire inserted into the angular-stable hole prior to enabling dynamic compression.
- Consider potential rotational changes during screw insertion [19].

**Fig. 26:**
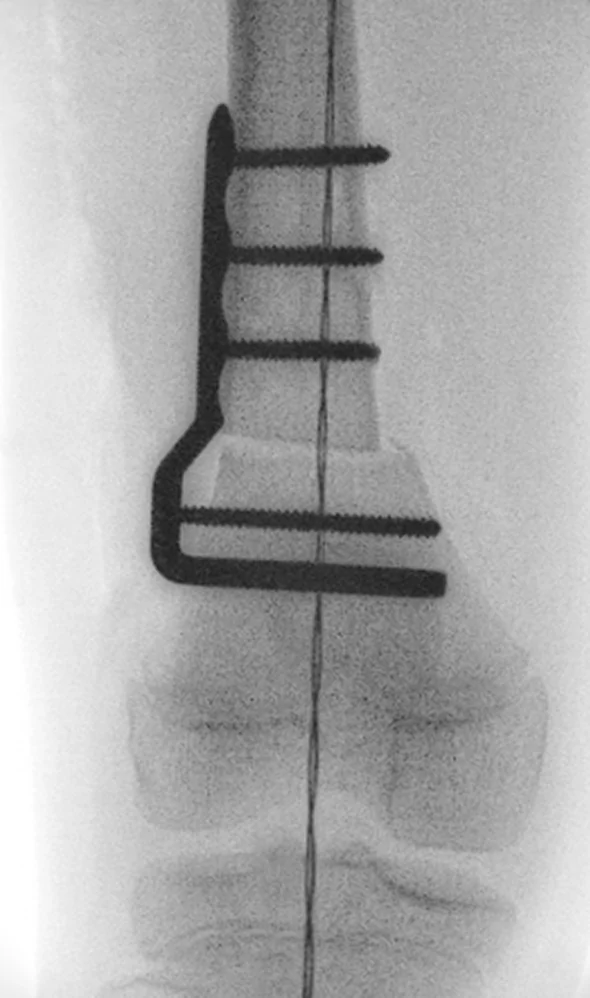
Final radiographic evaluation with projected leg axis

Once all screws are inserted (Fig. 25), a final clinical and radiographic evaluation is performed to conclude the procedure (Fig. 25, Fig. 26).

## Results

A total of 111 patients were included, with 77 in the Blade Plate group and 34 in the 90° LCP-PCP group. The mean age was 13.3 years (range 6.26–19.36) in the Blade Plate group and 13.9 years (range 7.15–19.52) in the 90° LCP-PCP group, with no statistically significant difference between groups (*p* = 0.740).

In the Blade Plate group, 34 patients (44%) were female compared with 9 patients (26%) in the 90° LCP-PCP group. The left side was affected in 36 patients (47%) in the Blade Plate group and in 14 patients (41%) in the 90° LCP-PCP group.

An underlying neurological disease was present in 62 patients (80%) in the Blade Plate group and in 20 patients (59%) in the 90° LCP-PCP group. Connective tissue disorders were rare in both groups, occurring in three patients (4%) and two patients (6%), respectively (Table 1).

**Table 1:** Patient characteristics

	Blade Plate group	90° LCP-PCP group	*p*-Value
Age	13.3 (3.24) (12.47–13.97)	13.9 (3.09) (12.83–14.85)	0.740
Female sex	34 (44%)	9 (26%)	0.131
Left side	36 (47%)	14 (41%)	0.359
Underlying neurological disease	62 (80%)	20 (59%)	0.017
Underlying connective tissue disorder	3 (4%)	2 (6%)	0.643

Mean (SD) (95% confidence interval) for age, and number (%) for all other variables

A total of 77 distal femoral osteotomies were performed using the PediLoc® Locking Cannulated Blade Plate System and 34 using the 90° LCP-PCP. One osteotomy performed using the Blade Plate had to be classified as a failure due to an intraoperative technical error that was not detected, which resulted in a fracture at the level of the inserted blade. This case was excluded from the subsequent data analysis. Consequently, 110 successful surgeries were included in the evaluation. Of the 76 surgeries that incorporated the Blade Plate, 13 were identified as pure extension osteotomies (17.1%), one as a varus osteotomy (1.3%) and 62 multidimensional osteotomies (81.6%). Among the 34 surgeries with the 90° LCP-PCP, 2 were pure extension osteotomies (5.9%), 4 pure varus osteotomies (11.8%), 1 pure valgus osteotomy (2.9%), 4 pure internal rotational osteotomies (11.8%) and 23 multidimensional osteotomies (67.6%).

Pre- and postoperative radiographic and clinical parameters are summarized in Table 2. Both groups showed significant improvement in femoral antetorsion and extension deficit postoperatively (Blade Plate: 22° → 13°, *p* < 0.001; 90° LCP-PCP: 24.5° → 13°, *p* < 0.001; extension deficit 15°/10° → 0°, *p* < 0.001). In the Blade Plate group, mLDFA increased slightly (87° → 87.5°, *p* = 0.041), whereas mLDFA and mMPTA remained unchanged in the 90° LCP-PCP group (87° → 87°, *p* = 0.806; 87° → 87°, *p* = 1.000, respectively).

**Table 2:** Comparison pre- and postoperatively for the Blade Plate group and the 90° LCP-PCP group

	Blade Plate	Blade Plate	*p*-Value
	Preoperative	Postoperative	
mLDFA (°)	87 (5) (84.76–87.40)	87.5 (1) (87.31–87.76)	**0.041**
mMPTA (°)	88 (2) (87.45–88.04)	88 (2) (87.45–88.04)	1.000
Femoral antetorsion (°)	22 (18) (18.06–25.68)	13 (3) (13.28–14.56)	** < 0.001**
Extension deficit (°)	15 (10) (12.10–17.25)	0 (0) (0.−0)	** < 0.001**

	90° LCP-PCP preoperative	90° LCP-PCP postoperative	*p*-Value
mLDFA (°)	87 (3) (85.43–88.98)	87 (1) (87.01–87.64)	0.806
mMPTA (°)	87 (1) (86.84–87.63)	87 (1) (86.84–87.63)	1.000
Femoral antetorsion (°)	24.5 (24) (20.61–31.22)	13 (3) (12.64–14.12)	** < 0.001**
Extension deficit (°)	10 (20) (7.20–14.56)	0 (0) (0–0)	** < 0.001**

Median (interquartile range)

Comparison between groups (Table 3) revealed no significant differences in any preoperative parameter (mLDFA, mMPTA, femoral antetorsion or extension deficit; all *p* > 0.05), confirming that the two groups were comparable before surgery. Postoperative comparisons also showed no statistically significant differences between groups (mLDFA *p* = 0.257; mMPTA *p* = 0.056; femoral antetorsion *p* = 0.473; extension deficit *p* = 1.000), with weak correlations between group assignment and these parameters (all r < 0.26). These findings demonstrate that both fixation methods achieve comparable radiographic correction and functional improvement (Table 4).

**Table 3:** Comparison between Blade Plate and 90° LCP-PCP groups (pre- and postoperative parameters)

	Preoperative *p*-Value	Preoperative r/*p*	Postoperative *p*-Value	Postoperative r/*p*
mLDFA (°)	0.805	0.241/0.200	0.257	0.245/0.191
mMPTA (°)	0.056	0.155/0.414	0.056	0.155/0.414
Femoral antetorsion (°)	0.258	0.260/0.165	0.473	0.145/0.446
Extension deficit (°)	0.087	−0.115/0.546	1.000	–

**Table 4:** Comparison of consolidation rates between both groups

	Score	Blade Plate group	90° LCP-PCP group	Percentage (%) above threshold (blade plate versus 90° LCP-PCP)	*p*-Value	Correlation r/*p*
6 weeks	REBORNE score	9 (2) (8.36–9.34)	9 (3) (8.11–9.69)	23% versus 19%	0.950	0.090/0.654
	RUST score	9 (2) (8.47–9.38)	8 (1) (7.29–8.25)	39% versus 6%	0.002	0.155/0.439
	mRUST score	9 (2) (8.23–9.26)	9 (2) (8.15–9.59)	0% versus 0%	0.796	0.298/0.132
3 months	REBORNE score	14 (3) (12.95–14.19)	14 (4) (12.96–14.61)	87% versus 96%	0.877	−0.140/0.566
	RUST score	12 (0) (11.18–11.80)	11 (3) (9.75–11.12)	91% versus 70%	< 0.001	0.155/0.439
	mRUST score	14 (3) (12.79–14.00)	14 (4) (12.20–14.06)	70% versus 61%	0.563	−0.149/0.542

Median (interquartile range) (95% confidence interval)

At the 6-week follow-up, imaging was available for 66 of 76 cases (87%) in the Blade Plate group; radiographs were missing in ten patients. In the 90° LCP-PCP group, imaging was available for 31 of 34 cases (91%), with three radiographs missing.

Median REBORNE scores were comparable between groups (Blade Plate: 9 [IQR 2]; 90° LCP-PCP: 9 [IQR 3]; *p* = 0.950), with 23% versus 19% of cases exceeding the predefined consolidation threshold.

The RUST score was significantly higher in the Blade Plate group (9 [IQR 2] versus 8 [IQR 1]; *p* = 0.002), and a greater proportion of patients exceeded the consolidation threshold (39% versus 6%).

The modified RUST score did not differ significantly between groups (9 [IQR 2] versus 9 [IQR 2]; *p* = 0.796), and no patient in either group reached the defined consolidation threshold (0% versus 0%). Correlation analyses demonstrated only weak associations between implant type and score values (all *r* ≤ 0.30).

At the 3-month follow-up, imaging was available for 53 of 76 cases (70%) in the Blade Plate group, with 23 radiographs missing. In the 90° LCP-PCP group, imaging was available for 23 of 34 cases (67%), and 11 radiographs were unavailable.

Both groups showed marked radiographic progression of consolidation. Median REBORNE scores were identical (14 [IQR 3–4]; *p* = 0.877), with consolidation rates of 87% in the Blade Plate group and 96% in the 90° LCP-PCP group.

The RUST score remained significantly higher in the Blade Plate group (12 [IQR 0] versus 11 [IQR 3]; *p* < 0.001), with 91% of patients exceeding the consolidation threshold compared with 70% in the 90° LCP-PCP group.

The modified RUST score showed no statistically significant difference between groups (14 [IQR 3] versus 14 [IQR 4]; *p* = 0.563), with consolidation rates of 70% versus 61%, respectively. Correlation coefficients again indicated weak associations between implant type and radiographic outcome measures.

## Discussion

The Distal PediLoc^®^ Locking Cannulated Blade Plate System is a specialized osteosynthetic implant system that is primarily used in paediatric orthopaedics for corrective osteotomies and treatment of femoral fractures.

The indications for corrective osteotomies encompass a wide range of conditions, including multidimensional deformities, rotational deformities, varus or valgus deformities and flexion contractures of the knee, particularly in patients with neurological conditions. However, in the context of such invasive surgical procedures, which are associated with a range of potential complications, including the intraoperative fracture that has been documented, these indications must be subjected to rigorous evaluation. It is imperative that symptoms such as pain, discomfort, gait disturbances and care-related issues, including transfer and body hygiene, are incorporated into the decision-making process. Furthermore, a comprehensive range of conservative interventions should be considered, including physiotherapy aimed at enhancing knee extension and less invasive surgical techniques such as tenotomies. Since the natural course of neurological conditions and severe deformities is frequently progressive, with deterioration of gait, motor function and pain being common consequences, corrective osteotomies are frequently the sole possibility for maintaining, or even ameliorating, the functional status of patients.

The device has been designed for application in growing bone structures. It integrates angular-stable locking technology with a cannulated shaft and blade plate configuration, thereby providing notable stability and precise placement [20]. The cannulated design facilitates optimal control of fragment positioning throughout the procedure, particularly in osteopenic bone and small anatomical structures, rendering it particularly advantageous for younger patients with underlying neuromuscular diseases [21].

The Blade Plate’s design is intended to ensure secure bone contact along the entire plate length. In addition, it is hypothesized that the design reduces bone stress, promotes even load transfer and minimizes complications [2, 3, 5, 6]. Biomechanical studies have shown that thread-guided screw holes in the proximal segment – such as those featured in the PediLoc® Locking Cannulated Blade Plate system – increase axial stability and tensile strength up to 12-fold in comparison with standard blade plates [22]. The plate’s multidirectional stability facilitates the absorption of loads from multiple orientations. This property offers a distinct advantage for active patients, as targeted microloading initiates callus formation, thereby accelerating the healing process [3, 5, 6, 23].

The assessment of osteotomy healing was conducted using REBORNE [24, 25], RUST [26] and modified RUST scores [15, 16], which are recognized in the scientific literature as reliable indicators for evaluating bone healing [13–18]. Our application of the Blade Plate System demonstrates healing rates comparable to the 90° LCP-PCP system within the first months after distal femoral osteotomy in children, based on the REBORNE and modified RUST score and a significantly higher consolidation rate in the RUST score at 6 weeks and 3 months postoperatively. The continuous increase in scores reflects progressive radiographic consolidation over time.

In accord with our results, Rutz et al. reported high stability and successful consolidation rates when using the 90° LCP-PCP. Their findings demonstrated that rapid mobilization and solid bone healing were achievable, thereby supporting outcomes similar to those observed in our data [27]. Thus, our findings indicate a significantly higher consolidation rate according to the RUST score in the Blade Plate group at both 6 weeks and 3 months. However, this difference was not observed using the REBORNE or modified RUST scores, suggesting that the advantage may be score-dependent rather than reflecting a universally superior healing response. The findings of this study are corroborated by additional research, which lends further support to the efficacy of the Blade Plate design. This design has been shown to promote consistent healing without the occurrence of implant failure, thereby underscoring the robustness of these systems and aligning with the stable consolidation values observed in the study [10, 12, 20, 28].

Aroojis et al. discussed distal femoral osteotomies using the L90° LCP-PCP and patellar tendon advancement as a therapeutic modality for patients diagnosed with cerebral palsy, with a particular focus on addressing crouch gait as a primary outcome. Their study demonstrated significant improvements in knee extension and muscle function, as well as a low complication rate [29]. While this study did not involve patellar tendon advancement and did not focus on treating crouch gait, the findings by Aroojis et al. highlight the importance of plate stability and multidimensional correction in improving functionality. These observations are comparable to the success of our multidimensional osteotomies, which were performed in 81.6% of cases, emphasizing the versatility of the distal Blade Plate system.

While studies such as those by Stout et al. (Synthes 90° blade-plate) and Brunner et al. (Depuy Synthes LCP Pediatric Condylar plate) did not use specific assessment scores, they also reported complete healing and stability within 12 weeks following corrective osteotomies of the distal femur [30–32]. The validity of these findings is further reinforced by the findings of subsequent studies [1, 8, 33]. These results are consistent with our 3-month consolidation rate, which has been objectively documented using REBORNE and RUST scores. The results demonstrate that the distal blade plate ensures reliable consolidation in complex distal femoral osteotomies, with comparable radiographic outcomes to the 90° LCP-PCP system in most evaluated parameters.

Prior to drawing definitive conclusions, it is imperative to consider the study’s strengths and limitations. The present study is subject to several limitations. Firstly, its retrospective design, devoid of randomization, engenders the risk of bias and confounding factors. For instance, disparities in indication may have exerted an influence on implant selection. Furthermore, the integrity of the imaging data was compromised at specific time points, thereby compromising the validity of interim analyses (e.g. 10 missing cases at the 6-week follow-up in the Blade Plate group; 10/76, 13%). The single-centre design limits the generalizability of the findings, and the unequal distribution of osteotomy types between the groups represents a potential source of bias (multidimensional osteotomies in 62/76 cases [81.6%] in the Blade Plate group; no detailed data available for the control group). The relatively limited sample size, in conjunction with the disparity in sample size between the two groups, has the potential to act as an additional confounding factor, thereby compromising the robustness of the findings.

It is important to acknowledge additional potential confounders that could have influenced our results. Firstly, the unequal distribution of underlying neurological conditions between the groups (80% in the Blade Plate group versus 59% in the 90° LCP-PCP group) may have affected postoperative consolidation rates, as patients with neuromuscular disorders often exhibit altered bone quality and healing dynamics [34, 35]. Secondly, the proportion of multidimensional osteotomies differed between groups (81.6% in the Blade Plate group versus 67.6% in the control group), which could impact mechanical stability and radiographic consolidation independent of implant type.

Thirdly, the missing radiographs at 3-month follow-up (30% of cases in the Blade Plate group versus 33% of cases in the 90° LCP-PCP group) are relevant and reduce the robustness of our results. Lastly, variations in preoperative deformity severity, although statistically nonsignificant, might have contributed to subtle differences in biomechanical loading and healing trajectories. These factors underscore the need for cautious interpretation and highlight the importance of prospective, randomized studies to minimize confounding.

Nevertheless, the study offers several strengths. It provides a direct comparison between two widely used implant systems for distal femoral osteotomies in a paediatric population. Bone healing was assessed objectively using validated scores (REBORNE, RUST, modified RUST), applied at clearly defined follow-up intervals (6 weeks and 3 months). The high proportion of complex, multidimensional osteotomies in the Blade Plate group (62/76, 81.6%) highlights the implant’s applicability in challenging clinical scenarios with high demands on stability.

The PediLoc® Blade Plates design is engineered especially for complex, multidimensional femoral osteotomies. Our data show reliable consolidation rates comparable with those of other plate designs in REBORNE scores and modified RUST scores and faster consolidation rates in RUST scores. Further studies are required to determine whether these findings translate into clinically relevant advantages such as earlier mobilization or shorter hospital stay, especially in children with neurological or complex orthopaedic conditions.

Hypothetically, earlier osteotomy consolidation would permit earlier initiation of weight-bearing and accelerated rehabilitation. Early mechanical loading stimulates osteogenesis and promotes functional recovery while simultaneously mitigating disuse-induced muscle atrophy [36, 37]. In patients with neuromuscular impairment, early mobilization is particularly critical, as prolonged immobilization exacerbates muscle weakness, impairs motor relearning and increases the risk of long-term functional decline [38, 39].

Therefore, earlier restoration of motor function and gait is a key determinant of long-term outcomes in this population, contributing to improved functional independence, reduced complication rates (e.g. contractures) and enhanced overall quality of life. However, even though the Blade Plate group showed faster osteotomy healing according to the RUST score, correlation analyses revealed only weak associations between implant type and healing scores. Our results are, due to the retrospective design and the several limitations, not robust enough to support earlier weight-bearing. Thus, clinical benefit, if present, might be marginal. While our data demonstrate reliable radiographic consolidation, further studies are required to determine whether these findings are related to the plates design or may be partly confounded by the differences between our studied groups. Connective tissue diseases, sex and side showed no statistical difference. The prevalences of neurological diseases and osteotomy complexity were unevenly distributed, which might represent relevant confounding factors.

In the PediLoc® Blade Plate group, the exclusion of one patient was necessitated by the misplacement of the chisel and the blade plate, resulting in an intraoperative fracture. To assess consolidation rates and compare the two different plate designs in osteotomies, it was necessary to exclude the patient from further analysis. Nevertheless, the purpose of highlighting this case is to raise awareness of this devastating intraoperative complication.

## Conclusions

This retrospective single-centre study provides preliminary evidence that the PediLoc® Blade Plate represents a stable and effective implant option for distal femoral osteotomies in paediatric patients, particularly in cases requiring complex, multidimensional correction. The observed tendency towards earlier radiographic consolidation and the high rate of bone healing suggest potential clinical benefits, including earlier mobilization and rehabilitation.

However, due to the retrospective design, lack of randomization, potential confounding factors, the high rate of missing radiographs at the 3 months follow-up and the limited generalizability of a single-centre setting, these findings should be interpreted with caution.

Further prospective, randomized and multicentre studies are required to validate these observations and to more robustly determine the comparative effectiveness and safety of different plate systems in paediatric distal femoral osteotomies.

## Data Availability

The datasets used and/or analysed during the current study are available from the corresponding author on reasonable request.

## Abbreviations

90° LCP-PCP: Synthes^®^ 90° Locking Compression Pediatric Condylar Plate
Blade Plate: PediLoc^®^ Locking Cannulated Blade Plate System
CT: Computed tomography
k-wire: Kirschner wire
MRI: Magnetic resonance imaging
mRUST: Modified Radiographic Union Score for Tibial Fractures score
RUST: Radiographic Union Score for Tibial Fractures score
